# The Potential of Intertwining Gene Diagnostics and Surgery for Mitral Valve Prolapse

**DOI:** 10.3390/jcm12237441

**Published:** 2023-11-30

**Authors:** Jasper Iske, Maximilian J. Roesel, Nikola Cesarovic, Leonard Pitts, Annabel Steiner, Leonard Knoedler, Timo Z. Nazari-Shafti, Serdar Akansel, Stephan Jacobs, Volkmar Falk, Joerg Kempfert, Markus Kofler

**Affiliations:** 1 Department of Cardiothoracic and Vascular Surgery, Deutsches Herzzentrum der Charité (DHZC), 13353 Berlin, Germany; jasper.iske@dhzc-charite.de (J.I.); maximilian.roesel@charite.de (M.J.R.); nikola.cesarovic@hest.ethz.ch (N.C.); leonard.pitts@dhzc-charite.de (L.P.); timo.nazari-shafti@charite.de (T.Z.N.-S.); serdar.akansel@dhzc-charite.de (S.A.); stephan.jacobs@dhzc-charite.de (S.J.); volkmar.falk@dhzc-charite.de (V.F.); joerg.kempfert@dhzc-charite.de (J.K.); 2 Charité—Universitätsmedizin Berlin, Corporate Member of Freie Universität Berlin and Humboldt-Universität zu Berlin, 10117 Berlin, Germany; 3 Berlin Institute of Health, 10117 Berlin, Germany; 4 Department of Health Sciences and Technology, ETH Zuerich, 8092 Zuerich, Switzerland; 5 Synlab MVZ, Humangenetik Muenchen, 80337 Muenchen, Germany; annabel.steiner@synlab.com; 6 Department of Plastic, Hand and Reconstructive Surgery, University Hospital Regensburg, 93053 Regensburg, Germany; lknoedler@mgh.harvard.edu; 7 BIH Center for Regenerative Therapies (BCRT), Berlin Institute of Health at Charité-Universitätsmedizin, Berlin, 13353 Berlin, Germany; 8 DZHK (German Centre for Cardiovascular Research), Partner Site Berlin, 10785 Berlin, Germany; 9 Berlin Institute of Health, Charité—Universitätsmedizin Berlin, 10117 Berlin, Germany

**Keywords:** mitral valve prolapse, minimally invasive surgery, genetic predisposition, minimally invasive mitral valve reconstruction, DZIP1, TNS1, filamin A

## Abstract

Mitral valve prolapse (MVP) is common among heart valve disease patients, causing severe mitral regurgitation (MR). Although complications such as cardiac arrhythmias and sudden cardiac death are rare, the high prevalence of the condition leads to a significant number of such events. Through next-generation gene sequencing approaches, predisposing genetic components have been shown to play a crucial role in the development of MVP. After the discovery of the X-linked inheritance of filamin A, autosomal inherited genes were identified. In addition, the study of sporadic MVP identified several genes, including DZIP1, TNS1, LMCD1, GLIS1, PTPRJ, FLYWCH, and MMP2. The early screening of these genetic predispositions may help to determine the patient population at risk for severe complications of MVP and impact the timing of reconstructive surgery. Surgical mitral valve repair is an effective treatment option for MVP, resulting in excellent short- and long-term outcomes. Repair rates in excess of 95% and low complication rates have been consistently reported for minimally invasive mitral valve repair performed in high-volume centers. We therefore conceptualize a potential preventive surgical strategy for the treatment of MVP in patients with genetic predisposition, which is currently not considered in guideline recommendations. Further genetic studies on MVP pathology and large prospective clinical trials will be required to support such an approach.

## 1. Introduction

With a prevalence of 2% to 4%, mitral valve prolapse (MVP) is a frequent valvular heart disease typified by chordal elongation or rupture leading to the left atrial displacement of segments of one or both mitral valve leaflets [1]. Although the majority of MVPs are benign, a significant proportion of patients will develop severe mitral regurgitation (MR), which—if left untreated—can lead to heart failure [2,3]. Several studies have demonstrated an association between MVP and ventricular arrhythmias, as well as sudden cardiac death [4,5].

In clinical practice, 2D transthoracic echocardiography (2D TTE) constitutes the gold standard for the evaluation of valvular heart disease, including MVP, and allows the early non-invasive assessment of the etiology and mechanism [6]. Doppler techniques, in turn, provide an accurate assessment of mitral regurgitation severity [7]. Although the accepted MVP definition is based on 2D TTE [8], three-dimensional transesophageal echocardiography allows for a more detailed analysis and better characterization of the affected segments [9]. Magnetic resonance imaging (MRI) can be used to quantify LV volumes and function as well as to determine the degree of subendocardial fibrosis as a marker of remodeling, which is often associated with MVP. The extent of fibrosis may be an important modifier for the arrhythmia risk in patients with MVP [10].

Fibro-myxomatous alterations of the valvular and chordal tissue have been characterized as the major pathologic correlate of MVP [11]. A distinction for MVP is made between fibro-elastic deficiency (FED) and Barlow’s disease (BD), which represent two different entities with regards to the changes in the extracellular matrix (ECM) [12].

FED can arise from various deficiencies in ECM proteins, including collagen, elastin, and proteoglycans, promoting the thinning of the leaflet and chordae. The larger surface area and atrialization of billowing leaflet segments increase the mechanical load on the attached chordae. This and the weakening of the degenerated chordal tissue increase the risk of chordal rupture. While most of the leaflet tissue is typically thin, myxomatous degeneration can also lead to local thickening, mainly in segments of the posterior leaflet [13]. Of note, patients frequently present at an older age with chordal rupture and flail after a rather short clinical history [14].

BD, in turn, is characterized by pronounced annular dilatation, bileaflet prolapse, billowing and hooding in the presence of valvular tissue redundancy and thickening due to excessive myxoid infiltration, and valvular and annular calcification of various degrees [13]. Patients are usually young or middle-aged, more frequently female, and often have a long history of heart murmurs and/or MR [14].

Notably, both the familial clustering of MVP in syndrome-associated conditions and familial patterns, as well as sporadic forms, suggest that predisposing genetic components play a crucial role in the development of MVP, which has prompted focused genetic research [15,16]. Recent data identified a plausible link between arrhythmogenicity and familial predisposition in patients with MVP [17,18].

The gold standard for the treatment of MVP is surgical mitral valve repair (MVR), which can be performed at very low risk with excellent short- and long-term results [19]. Current guidelines therefore recommend MVR at an early stage of the disease and before the occurrence of significant LV remodeling [20,21,22]. Complex mitral valve pathologies involving multiple segments should be treated in high-volume heart valve centers that can achieve high repair rates and excellent clinical outcomes [23,24]. Minimally invasive mitral valve surgery (MIVS) through a mini-thoracotomy and using endoscopic guidance appears to be non-inferior, if not better, compared to conventional median sternotomy (MS) in this context and is associated with fewer perioperative complications [25].

With significant progress in the development of next-generation genetic screening tools and the availability of minimally invasive surgery techniques, preventive rather than curative treatment approaches for MVP at early stages of the disease come into focus. In this review, we will discuss known gene mutations and their pathophysiological role in MVP, outline their potential role for screening and identifying patients at risk, and critically discuss strategies to eventually implement a preventive surgical approach based on genetic profiling.

## 2. X-Linked Heritage

Several studies have attempted to define disease-promoting genes involved in the pathogenesis of MVP. Initial approaches aimed to determine gene loci associated with familiar hereditary MVP, which can be inherited both autosomal-dominantly and through x-linked inheritance, by studying larger families with hereditary MVP. An x-linked recessive form of MVP was the first mutation found [26].

A standard positioning cloning approach in a large family affected by XMVD led to the discovery of a P637Q mutation in the FLNA gene encoding for Filamin A [27], an actin-binding protein that mediates cell motility and membrane stability [28]. In addition, Filamin A is broadly involved in cardiac development, particularly in fetal heart valve morphogenesis [29]. Investigations in smaller, unrelated families identified two other missense mutations (G288R and V711D) and a 1944-bp genomic deletion coding for exons 16 to 19 in the *FLNA* gene [27].

Mechanistically, Filamin A has been conceptualized to promote the development of myxomatous changes in valve tissue by the regulation of transforming growth factor-β (TGF-β) signaling through its interaction with SMAD transcription factors activated by TGF-β receptors [30]. In support of this, defective signaling cascades that involve members of the TGF-β superfamily have been described in the impaired remodeling of cardiac valves during development [31]. However, in a follow-up study searching for TGFBR1 or TGFBR2 mutations in families with isolated familial cases of MPV, neither TGFBR1 nor TGFBR2 mutations were detected, suggesting that they play a minor role in isolated myxomatous valvular dystrophy [32]. Notably, in vitro studies investigating two FlnA–G288R and P637Q mutations in human melanoma and fibrosarcoma cell lines delineated an altered balance of the small GTPases RhoA and Rac1 involved in the actin cytoskeleton during cell adhesion, with spreading and migration providing a potential mechanism contributing to the pathogenesis of MVP [33]. A subsequent study examining the outlined mutations in a yeast two-hybrid screen confirmed altered cell–ECM crosstalk, focal adhesion transduction pathways, and actin cytoskeleton dynamics through impaired PTPN12 signaling [34].

## 3. Autosomal-Dominant Heritage

The examination of larger families with an autosomal-dominant MVP trait resulted in the identification of three loci for genetic myxomatous MVP, with MMVP 1 being located on chromosomes 16p11.2–p12.1 [35], MMVP2 on chromosome 11p15.4 [36], and MMVP 3 on chromosomes 13q31.3–q32.1 [37]. Notably, for MMVP2 alone, the reflective gene, namely DCH1s, was found to display missense variants (p.R2513H and p.R2330C) [38], while the reflective genes and proteins for MMVP1 and MMVP 3 remain elusive. Further genetic studies identified two additional families in which a second deleterious DCHS1 mutation was segregated with MVP. Mechanistically, both DCHS1 mutations appear to impair the stability of the encoded signaling peptide of the cadherin family, which mediates planar cell polarity, as demonstrated by morpholino knockdown in zebrafish as well as human mitral valve interstitial cells. Strikingly, DCHS1^+/−^-knockout mice defective for DCHS1 developed MVP with thickened valve leaflets [38]. Sequencing all coding regions of DCHS1 in a follow-up study revealed eight missense variants including six considered deleterious by in silico prediction analysis. These included one novel variant (p.A2464P) and two rare variants (p.R2770Q and p.R2462Q). Notably, rare in silico predicted pathogenic variants were also frequently identified in sporadic cases of MVP in DCHS1 [39].

The parallel development of modern genome-wide association studies (GWASs) has paved the way for the characterization of further genes involved in MVP, leading to the discovery of several targets involved in both hereditary and non-hereditary MVP.

## 4. Sporadic MVP

### 4.1. DZIP1

DZIP1 has been characterized as contributing to both hereditary and non-hereditary MVP. Indeed, the whole-exome sequencing of individuals suffering from MVP in a family displaying autosomal-dominant inheritance patterns of MVP revealed a single heterozygous missense variant in the DZIP1 gene [40]. Mechanistically, DZIP1 mutations impair the stability of a multimeric complex consisting of DZIP1, CBY1, and beta-catenin. Thus, the suppressive activity of DZIP1/CBY1 on beta-catenin, which has been delineated as promoting endothelial-to-mesenchyme transformation, endocardial proliferation, ECM remodeling, and the myxomatous degeneration of valves [41,42], is impeded. This leads to the augmented activation of its target gene MMP2, ultimately promoting the loss of the collagenous ECM matrix and resulting in a myxomatous phenotype in the mitral valve [43]. Notably, a mouse model harboring this variant confirmed the pathogenicity of the DZIP1 mutation and revealed impaired ciliogenesis during development, which progressed to adult myxomatous valve disease and functional MVP [40].

### 4.2. TNS1

An initial discovery meta-analysis of two independent French GWASs revealed the overall strongest association on Chr2q35 in a ~424 Kb gene-desert region, with TNS1 being one of the closest downstream gene loci [44]. TNS1 encodes for Tensin1, which takes part in focal adhesion control involving actin binding [45]. During murine valve morphogenesis, Tensin1 was found to be expressed in endothelial and valvular interstitial cells, persisting during adulthood. Notably, Tensin1-knockout mice displayed enlarged posterior mitral leaflets with myxomatous degeneration associated with slight leaflet displacement in echocardiographs, indicating its involvement in the development of myxogenous MVP. The functional significance of this impairment was subsequently confirmed in zebrafish experiments, revealing increased AV regurgitation in TNS1 morpholino knockdown. Moreover, the expression of bmp4, a pattern of valve development, was significantly altered, indicating TNS1 involvement during cardiac development. Of note, Filamin A, which has been associated with rare X-linked forms of MVP, exerts a similar function of mediating actin binding [27].

### 4.3. LMCD1

In a global meta-analysis that included the French GWAS discovery study and follow-up datasets from European American and European Spanish cases and controls, the strongest association on Chr3p13 was observed for rs171408, which maps to an intron of LMCD1, a transcript of the LIM domain family of zinc finger proteins [44]. Specifically, the increased expression of LMCD1 in mouse hearts was found to directly suppress GATA6, which is involved in cardiac development [46]. Indeed, morpholino knockdown in zebrafish resulted in increased AV regurgitation, while the morphological analysis of the developing myocardium revealed that LMCD1 morphants exhibited a modest reduction in cardiac looping [44]. Of note, the expression of notch1b and bmp4 was not altered, indicating that LMCD1 dysfunction more likely impairs general cardiac development associated with MVP.

### 4.4. GLIS1

An improved Gene Set Enrichment Analysis, in which enrichment was calculated based on all association results of the GWAS meta-analysis without limiting them to a significance threshold [47], in conjunction with subsequent array-based human transcriptomic data from tissues and cell types, was performed. It revealed another mutation, 1p32.3, in GLIS family zinc finger 1 (GLIS1) located on chromosome 1, encoding the GLI-related Kruppel-link zinc finger protein transcription factor [48]. The translational relevance of this protein for MVP was subsequently delineated by immunohistochemistry analysis during different development stages of the murine heart and zebra fish morpholino knockdown. Strikingly, GLIS1 was predominantly expressed in the nuclei of endothelial and valvular interstitial cells during embryonic development, with a significant decline in the hearts of adult mice. Consistently, knockdown in zebrafish induced atrioventricular regurgitation [48].

### 4.5. PTPRJ and FLYWCH

A genome-wide linkage analysis in a four-generation pedigree with several family members exhibiting severe MVP revealed several significant regions associated with MVP. The subsequent alignment of the linkage analysis results with whole-exome sequencing in the most affected family member and subsequent segregation analysis revealed two heterozygous missense variants, FLYWCH1 p.R540Q on chr. 16p13.3 and PTPRJ p.I1013S on chr. 11p11.2, present in most affected family members while absent in all unaffected family members. Notably, the expression of PTPRJ and FLYWCH was confirmed in cultures of human MV cells by qPCR [49]. Supporting the concept of PTPRJ’s involvement in the development of MPV, this receptor tyrosine phosphatase involved in ERK signaling seems to mediate the early development of cardiac valves, while mice homozygous for a null allele exhibited endocardial cushion defects and broad defects in endothelial cell signaling [50]. Similarly, FLYWCH1, a FLYWCH-type zinc-finger-containing protein, has been characterized as a potential master regulator of artherogenesis that is potentially of translational relevance for valve development [51].

### 4.6. MMP2

Myxomatous tissue dysfunction has been linked to the increased release of metalloproteinases (MMP), and an inverted decrease in tissue inhibitors of metalloproteinases (TIMP) responsible for the degeneration of collagen and elastin structures has been observed [52,53,54]. More importantly, augmented systemic MMP levels in patients suffering from mitral valve disease (MVD) with both stenosis and regurgitation have been described [52,55]. Comparing the genotype distributions and allele frequencies of MMP-SNPs between patients affected by MVD and healthy controls revealed a significant association between the occurrence of MMP-SNPs and MVD [56]. Moreover, the MMP2 rs243865 SNP has been closely correlated with the occurrence of MVP specifically [57].

### 4.7. SPTBN1

A meta-analysis of six genome-wide association studies confirmed previously identified gene loci and furthermore delineated two novel gene loci associated with MVP through the integration of epigenetic, transcriptional, and proteomic data. Moreover, for the first time, a polygenic risk score involving various gene loci was created with improved MVP risk prediction beyond age, sex, and clinical risk factors [58].

An interesting candidate was SPTBN1 [58], which encodes β2-spectrin, mediating the linkage of the actin cytoskeleton to the plasma membrane. MVP risk was thereby associated with a decreased expression of SPTBN1 in the human heart. β2-spectrin deficiency, in turn, has been shown to cause the dysfunction of the cytoskeleton and impair heart development [59]. These findings could indicate the involvement of β2-spectrin during heart development with the diminished expression of SPTBN1, leading to the disruption of the cytoskeleton during MV development.

### 4.8. ALPK3

In the same study, ALPK3 was linked to MVP [58], which has previously been associated with non-sarcomeric HCM and forms of dilated cardiomyopathy [60]. Notably, in HCM, both elongated mitral valve leaflets and the myxomatous degeneration of the valve have been described [61]. Identifying ALPK3 as a gene associated with MVP potentially underscores the convergence of the myopathic and valvular pathogenic pathways.

### 4.9. LTBP2

The latent transforming growth factor-beta-binding protein 2 (LTBP2) codes for an extracellular matrix protein that binds to microfibrils containing fibrillin-1, playing a role in regulating the signaling of TGF-β [58]. Additionally, LTBP2 has been linked to connective tissue disorders such as isolated ectopia lentis and Weill–Marchesani syndrome [62]. Initially, LTBP2 was associated with MVP in an unpublished study investigating a large family with a C-to-T nucleotide substitution (https://heartvalvesociety.org/meeting/abstracts/2016/P8.cgi) (accessed on 15 November 2023) This observation was confirmed in the outlined meta-analysis [58]. Moreover, another group who also described a rare LTBP2 Val1506Met mutation segregating with MVP in a family with hereditary MVP further investigated the mechanistic involvement utilizing a LTBP2-knockout study, in which a significant proportion of mice carrying the mutation demonstrated myxomatous valve degeneration [63].

## 5. Paving the Way for Screening Patients at Risk

Although MVP is mostly benign and often asymptomatic, MVP can cause severe MR and lead to atrial fibrillation (AF) and heart failure. Ventricular tachycardia with an increased risk for sudden cardiac death (SCD) is less frequent and predominantly affects young adult women [17,64,65,66,67,68,69]. Myocardial stretching by the prolapsing leaflet may induce the fibrosis of the papillary muscles and inferobasal left ventricular wall, which is thought to be the morphological substrate responsible for ventricular arrhythmias [18,70]. Notably, an early study investigating familiar MVP in a larger family provided evidence for an inherited structural phenotype associated with MVP-derived supraventricular tachycardia and SCD [71].

Subsequently, several molecular studies investigating disease-driver genes and molecular fingerprints of MVP have correlated several mutations with the clinical prognosis of MVP as well as the prevalence of related complications. A recent study reconciling the expression of MMPs and TIMPs in valves excised from patients undergoing cardiopulmonary bypass surgery for MVP demonstrated that the increased expression of MMPs in addition to compromised TIMP tissue levels is associated with the severity of MR [55]. Hence, the authors suggested the assessment of MMPs for the early diagnosis and therapy of MVP. Consistent with this finding, the occurrence of MMP SNPs has been shown to correlate with augmented systemic levels of MMPs as well as pro-BNP levels, which are associated with the severity of MVD as well as disease-related complications including pulmonary hypertension, tricuspid regurgitation, cerebrovascular disease, and heart failure [56].

These results provide evidence that genetic disposition could be utilized to identify a patient population at risk for future symptomatic MVP and related complications, including life-threatening ventricular arrhythmias. Supporting this concept, a recent study demonstrated that males carrying FLNA mutations exhibit a severe phenotype of MVP, often already manifesting at a young age with poly-valvular involvement and consecutive worsening over time, presenting a substantial lifetime risk (approx. 75%) of valve surgery in male patients at 70 years [72,73]. Similar observations have been made in patients with non-FLNA MVP [74] or Marfan syndrome [75]. Whole-exome autopsies in patients succumbing to unexplained SCD in their youth isolated MVP as the only abnormal postmortem finding, with three genes (p.E1518fsX25-DMD, p.S285N-RYR2, and p.R109X-TTN) linked to cardiomyopathy and channelopathy [76]. In a patient with bileaflet MVP and frequent multifocal PVCs and the affected maternal aunt, whole-exome sequencing identified a novel truncating variant in FLNC-encoded Filamin C (p.Trp34*-FLNC) as a potential proarrhythmic genetic substrate for arrhythmogenic bileaflet MVP syndrome [77]. Similarly, the most recent case report on a woman with bileaflet MVP and multiple types of arrythmias also demonstrated a frameshift and truncating pathogenic variant in FLNC [78]. These findings support the need for early genetic screening to identify patients at risk [76,79].

**Figure 1:**
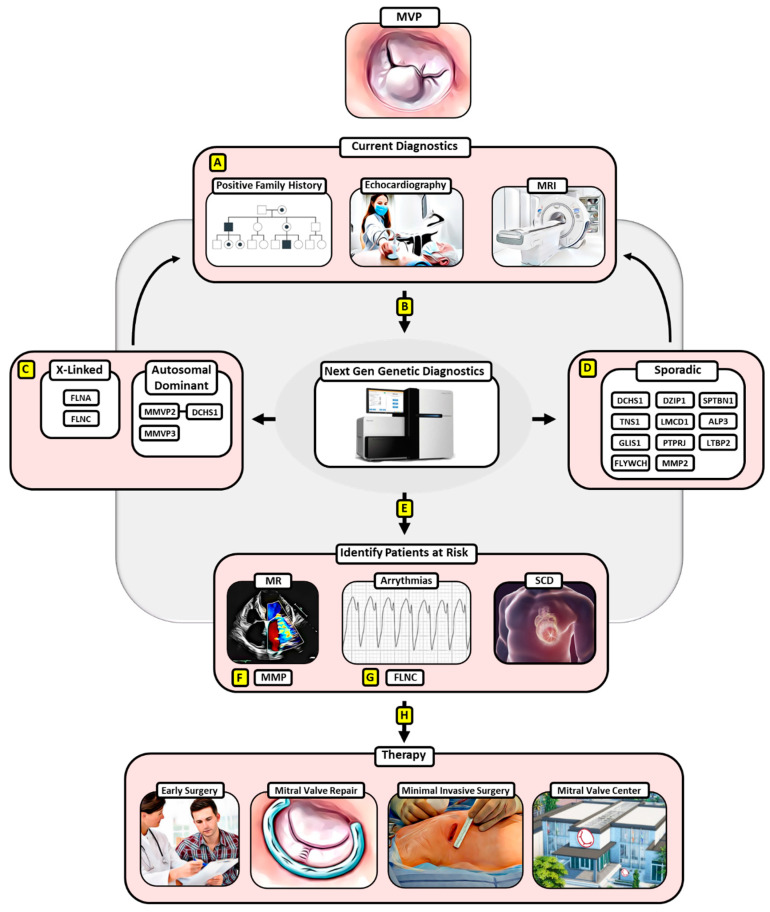
Potential algorithm for implementing next-generation genetic diagnostics and surgical treatment for MVP. (**A**) Current diagnostics involving a positive family history for MVP in the anamnesis, echocardiography, and MRI can be expanded by (**B**) next-generation genetic diagnostics that have enabled the detection of pathologic gene mutations for (**C**) hereditary MVP and (**D**) sporadic MVP, which in turn may improve diagnostic specificity. (**E**) Moreover, next-generation genetic diagnosis could enable the identification of MVP patients with a risk of adverse events involving MR, malign arrythmias, and SCD. (**F**) Thereby, MMPs have already been linked to the development of severe forms of MR in MVP, (**G**) while an FLNC-variant has been identified as a proarrhythmogenic genetic substrate of arrhythmic MVP. (**H**) MVP patients at risk according to genetic diagnostics can then be subjected to early surgical treatment favoring mitral valve repair via a minimally invasive approach at a designated mitral valve center, ensuring the best clinical outcomes. Abbreviations: MVP—mitral valve prolapse, MRI—magnetic resonance imaging, SCD—sudden cardiac death, MMPs—matrix metalloproteinases, FLNC—filamin c.

The increasing number of identified gene loci and disease-driving mutations in the pathology of MVP and the availability of easily accessible molecular screening tools will allow early diagnostics and eventually early intervention in families with an inheritance line for MVP as well as patients displaying MVP (Figure 1).

Therefore, expanding the diagnostics for MVP by collecting EDTA samples would be required to enable genetic testing. According to current diagnostic standards, whole-exome sequencing (WES) is performed, a cost-effective high-throughput technology, which allows the analysis of all protein-coding regions of the genome [80]. In order to detect disease-relevant variants, they are filtered based on the clinical symptoms of the patient, population frequency, current literature, possible influence on protein function, and mode of inheritance [2]. For some genes, clinically relevant variants must be confirmed by further methods such as Sanger sequencing [81]. In the case of a positive family history of MVP, the analysis of genes associated with familial clustering is carried out as the first step. If this first-step analysis does not provide any positive findings, the genes associated with sporadic MVP are analyzed in a second step. Conversely, in the case of a blank family history, genes associated with sporadic forms are analyzed first, and genes related to familial clustering follow in a second step for the detection of de novo variants. This step-by-step procedure ensures that the number of variants of unknown significance (VUS), which cannot be classified as either benign or pathogenic according to the current state of knowledge and the literature, is minimized [3]. Lastly, a qualified geneticist needs to manually examine the filtered candidates under the consideration of databases and the literature. Depending on the laboratory, the turnover time of a WES analysis is currently between several days and several weeks [1]. Based on the findings of the genetic screening, therapeutic strategies, such as an early surgical intervention, could be adapted according to the patient’s individual risk.

However, it must be taken into account that the current status of genetic research on MVP is based on small pedigrees and medium-sized case control studies, which have only led to the identification of a few genetic loci involved in MVP pathogenesis. Notably, these loci in turn only account for a small fraction of the interindividual genetic variability [16]. Considering the polygenic nature of MVP, it may be challenging to delineate further genetic factors, in particular those with relevance for malignant MVP, and further large-scale studies may be required [16]. Thus, the clinical relevance of the described findings and their translational relevance for current guidelines remain limited and are under investigation at the current time point.

## 6. Early Surgery for MVP May Provide a Treatment Option for at-Risk MVP Patients

There is no effective pharmacological treatment inhibiting the progression of MVP to MR or preventing the occurrence of adverse events (AEs), including malign arrhythmias or SCD. Although MVP, even when complicated by moderate or even severe mitral regurgitation, usually remains asymptomatic in its early stage, atrial and ventricular remodeling including fibrosis occur [82].

Even though still in an experimental stage, considering the progress of genetic analysis techniques and big data analysis, the genetic testing of patients with MVP may identify those at risk and inform future guidelines with regards to the indications and timing for surgical interventions. There is sufficient evidence for minimally invasive surgical MVP interventions with excellent results in both the short and long term [19,83].

Surgical mitral valve repair is the preferred treatment option for primary MR [21,84,85]. Current guidelines recommend MVR for patients with severe mitral regurgitation and symptoms, as well as for patients with a left ventricular (LV) end-systolic diameter > 40 mm (European [21] and American [22] guidelines) and/or an LVEF < 60% even if they are asymptomatic. Surgery should also be considered in asymptomatic patients with preserved LV function and AF or pulmonary hypertension (systolic pulmonary artery pressure at rest > 50 mmHg). Finally, surgery may be appropriate in asymptomatic patients with an LVEF > 60%, LVESD < 40 mm, and significant LA dilatation (volume index ≥ 60 mL/m^2^ or diameter ≥ 55 mm) if permanent repair is likely, the surgical risk is low, and the repair is performed in a heart valve center [21,86]. Subjecting asymptomatic patients with a genetically determined risk for MR development or AEs to MVR may thus constitute a feasible treatment approach, once molecular and genetic screening diagnostics can effectively identify those patients.

Early surgery has been shown to provide significantly better long-term outcomes in patients with MR in the case of feasible mitral valve repair. In an analysis of the large multicenter Mitral Regurgitation International Database (MIDA) including more than 1000 patients, early MVS led to a survival rate of 86% after ten years in patients with flail mitral valve leaflets and early surgery compared to 69% with initial pharmaceutical management in an unmatched group comparison [20]. Consistently, a propensity score matched analysis confirmed higher survival rates after early surgery (HR 0.52) [20]. More importantly, heart failure and cardiac death were more common in patients who did not receive surgery during an early disease stage [87]. Early mitral valve repair can prevent atrial fibrillation, pulmonary hypertension, or congestive heart failure, all of which have been linked to poorer outcomes [88]. Consistently, the short-term mortality rate is low in patients undergoing surgery who exhibit no or only mild symptoms, confirming the benefits of early surgery [89]. Several repair strategies for primary MR are available and can be tailored according to the underlying pathology [90].

A recent observational study investigating the Society of Thoracic Surgeons Adult Cardiac Surgery Database to evaluate the association between hospital- and surgeon-level volume and MVr outcomes demonstrated that centers with a high volume and therefore experienced surgeons recorded a decreased risk-adjusted 30-day mortality, lower 30-day composite mortality plus morbidity, and higher rates of successful MVr [24,91]. In contrast, smaller centers tended to favor valve replacement, although MVr was possible in most cases [92]. Therefore, preventive surgery for young MVP patients at genetic risk should only be implemented in ’heart valve centers of excellence’, whose establishment is currently being pursued by international cardiothoracic surgery societies [93].

## 7. Conclusions

Although progression to arrhythmia and SCD is rare in patients with MVP, the high prevalence of the disease does lead to a significant number of events. However, the molecular pathology of MVP is heterogenous and multi-factorial, which has initiated the identification of early diagnostics and targeted therapy approaches. Next-generation gene sequencing studies have paved the way for investigating molecular mechanisms and have achieved promising results, identifying a broad range of potential disease-driver genes. Given the excellent long-term results of early MVR when performed in a minimally invasive manner in heart valve centers, the introduction of gene diagnosis followed by surgical treatment constitutes a concept, though currently only in the investigational stage, with significant potential for the future considering the progressive development of genetic diagnostics and analysis techniques, which may prevent AEs in asymptomatic MVP patients. Therefore, the identification of further disease-driver genes involved in the pathology of MVP and their confirmation in large prospective clinical studies are required.
